# Determinants of private-sector antibiotic consumption in India: findings from a quasi-experimental fixed-effects regression analysis using cross-sectional time-series data, 2011–2019

**DOI:** 10.1038/s41598-024-54250-2

**Published:** 2024-02-29

**Authors:** Shaffi Fazaludeen Koya, Senthil Ganesh, Sakthivel Selvaraj, Veronika J. Wirtz, Sandro Galea, Peter C. Rockers

**Affiliations:** 1 School of Public Health, Boston University, Boston, MA USA; 2 Public Health Foundation of India, New Delhi, Delhi India

**Keywords:** Policy and public health in microbiology, Infectious diseases, Health care, Public health

## Abstract

The consumption of antibiotics varies between and within countries. However, our understanding of the key drivers of antibiotic consumption is largely limited to observational studies. Using Indian data that showed substantial differences between states and changes over years, we conducted a quasi-experimental fixed-effects regression study to examine the determinants of private-sector antibiotic consumption. Antibiotic consumption decreased by 10.2 antibiotic doses per 1000 persons per year for every ₹1000 (US$12.9) increase in per-capita gross domestic product. Antibiotic consumption decreased by 46.4 doses per 1000 population per year for every 1% increase in girls’ enrollment rate in tertiary education. The biggest determinant of private sector antibiotic use was government spending on health—antibiotic use decreased by 461.4 doses per 1000 population per year for every US$12.9 increase in per-capita government health spending. Economic progress, social progress, and increased public investment in health can reduce private-sector antibiotic use.

## Introduction

Antibiotics have increased life expectancy by reducing community spread of infections and reducing in-hospital mortality due to hospital acquired infections and peri-operative infections worldwide. However, bacterial infections continue to cause significant global morbidity and mortality particularly in low-and middle-income countries.

The consumption or use of antibiotics is not uniform across countries or between regions within a country. Existing global literature suggests that a broad range of factors influence antibiotic use besides infectious disease burden. These include perceptions and behaviors, health system factors, climate and environmental changes, economic productivity, government spending on health, literacy and education, and vaccination coverage.

First, many studies have described behavioral factors that influence antibiotic use including patients’ illness perception and health-seeking, practitioners’ perception on legal issues and practice context, and wider social norms and peer pressure^1–3^. Second, health system factors like the doctor-population ratio, resources and infrastructure, density of medical practices, patients’ experiences with the health system and physicians including physicians’ prescribing behaviors, and medication effectiveness and medicine side effects affect antibiotic use^4–6^. Third, climate and environmental changes have been shown to influence antibiotic use—through changes in infection incidence and increase in microbial resistance^1,4,7–9^. Fourth, numerous studies have shown that measures of economic productivity, particularly per-capita gross domestic product (GDP), are predictors of antibiotic use^1,10–15^. Fifth, government spending on health^5,9^, and subsequent effect on overall healthcare expenditure, and patients’ out-of-pocket expenditure^9,16^ determine antibiotic use. Sixth, literacy rate and schooling, especially women’s and girls’ higher education and empowerment^10,17–20^, play a significant role in determining the quantity and appropriateness of antibiotic use. Seventh, improved vaccination coverage decreases antibiotic use by reducing incidence of bacterial infections like Typhoid, Haemophiles influenza B and Pneumococci^21^, by reducing viral infections for which antibiotics are inappropriately taken (e.g. Seasonal Influenza), and by reducing secondary bacterial infections following vaccine preventable viral infections^22,23^. Finally, respiratory infection incidence is a good predictor of antibiotic use, as indicated by previous studies from India and the US^24,25^.

However, our understanding on these key drivers of antibiotic consumption is largely limited to observational studies. The only exception of which we are aware was a global analysis by Klein et al.^26^ which examined the role of economic growth, measles vaccination rate, imports, and physician density on any antibiotic use during 2000–2015 among low middle income countries (LMICs) and high income countries (HICs). Using a quasi-experimental method—the fixed effects design—that study reported a significant positive association between GDP per-capita and changes in the antibiotic consumption rate in LMICs but not in HICs, while other factors were not found to be significant in either group^26^.

Indian data—with substantial differences across states and changes over years—provide an opportunity to explore the key factors that drive antibiotic consumption. India is among the countries with the highest burden of mortality due to infectious disease. Diarrheal disorders, lower respiratory tract infections, tuberculosis, and childhood pneumonia were among the top ten causes of deaths in India in 2019^27^. Infectious disease burden varies across Indian states^28^, while health systems and services are not uniform^29^. India’s national health mission classifies states into high focus (HF) states and non-high focus (nHF) states based on health infrastructure, life expectancy, fertility rate, and child and maternal mortality indicators^30^. HF states include Bihar, Chhattisgarh, Jharkhand, Madhya Pradesh, Odisha, Rajasthan, Uttar Pradesh, and seven northeastern states. Our previous work has shown that antibiotic consumption differences across Indian states are as big as differences across countries, and that antibiotic consumption decreased in many Indian states from 2016 to 2019^31^. It is therefore important to understand the factors determining antibiotic consumption in India as this will help policy makers identify and invest in the most efficient and effective measures to reduce antibiotic consumption.

Among the eight groups of factors that we discussed above, the first three groups—related to perceptions and behaviors, health systems, and climate and environmental factors—are largely time invariant, meaning we do not expect substantial changes in these factors over a few years. Therefore, in this paper, we attempt to examine the effects of the remaining five time-varying factors described in the literature—namely economic productivity, government spending on health, girls’ higher education rate, measle vaccination rate, and lower respiratory tract infection incidence on private-sector antibiotic consumption. We do so by using annual state-level private sector antibiotic consumption—measured as defined daily doses (DDD) consumed per 1000 population per day (DID)—across Indian states from 2011 to 2019 using a quasi-experimental fixed effects regression method. In addition, we controlled for the potential confounding effect of some of the time invariant factors discussed above.

## Results

**Figure 1:**
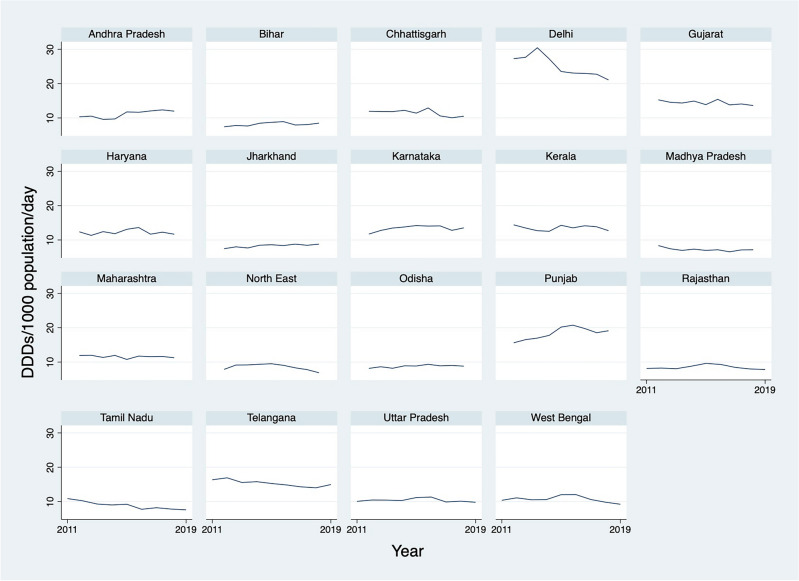
Time series of private-sector antibiotic consumption in Indian states, 2011–2019. *Note:* *DDD* defined daily doses.

The median private-sector antibiotic consumption across all the states for the entire study period was 11.2 DIDs (IQR = 4.9). The time series graphs of antibiotic consumption in 19 states from 2011 to 2019 are shown in Fig. 1. Across all the years, the highest annual value (30.5) was recorded in Delhi in 2013 and the lowest (6.6) was recorded in Madhya Pradesh in 2017. The median DID value was 10.9 (IQR = 6.3) in 2011, reached a maximum of 12.0 in 2016 (IQR = 4.9), and ended at the lowest value of 10.5 (IQR = 5.0) in 2019.

The summary of outcome and predictors and differences between HF and nHF states is given in Table 1. The median DID during the nine years was only 8.6 in HF compared to 13.1 in nHF states and Wilcoxon rank sum test showed that the difference was statistically significant (*p* < 0.0001).

**Table 1:** Private-sector antibiotic consumption (DID) and its predictors: overall median and inter-quartile ranges and differences between High Focus and non-High Focus States in India, 2011–2019.

Variables	Median [p25, p75]			
	Overall	Differences between state groups		
		High focus states (n = 8)	Non-high focus states (n = 11)	*p* value^a^
DID	11.2 [8.8, 13.7]	8.6 [8.0, 9.6]	13.1 [11.6, 15.1]	< 0.001
Per capita GDP, in ₹ ‘000 s, at current prices	108.5 [74.5, 160.1]	73.1 [55.2, 94.7]	148.1 [111.8, 194.0]	< 0.001
Per capita government spending on health, in ₹	988.3 [723.7, 1310.8]	808.0 [592.0, 1114.8]	1,101.9 [822.7, 1424.2]	< 0.001
Girls’ tertiary education enrollment, %	25.5 [17.6, 30.5]	18.1 [14.6, 23.1]	28.5 [25.0, 34.6]	< 0.001
Measles vaccination, per 1000 eligible children	907.0 [813.1, 970.8]	856.0 [799.8, 908.8]	960.0 [842.9, 995.0]	< 0.001
LRTI incidence, %	10.0 [9.0, 10.7]	10.6 [10.1, 11.0]	9.3 [8.4, 10.1]	< 0.001

*p25* 25th percentile, *p75* 75th percentile, *₹* Indian rupees, *DID* defined daily doses per 1000 population per day, *GDP* gross domestic product, *LRTI* lower respiratory tract infection.

^a^*p* values correspond to Wilcoxon rank sum tests for differences between high focus and non-high focus states.

The year-wise state-wise data are given in the Supplement. At the national level, the median per-capita GDP was ₹108,500 (IQR = 85,600), and it ranged from ₹21,800 in Bihar in 2011 to ₹376,000 in Delhi in 2019 (Supplement Table S2). The median per-capita government spending on health was ₹988.3 (IQR = 587.1) and it ranged from ₹302.7 for Bihar in 2011 to ₹3777.8 for Delhi in 2019 (Supplement Table S3). The median girls’ enrollment rate in tertiary education was 25.5% (IQR = 12.9), and the rate ranged from 9.5% in 2011 in Jharkhand to 51.8% in 2019 in Delhi. (Supplement Table S4) The median measles vaccination rate was 907.0 per 1000 eligible children (IQR = 157.7). This ranged from 737.3 for Tamil Nadu in 2016 to 1439.0 for Telangana in 2016 (Supplement Table S5). The median LRTI incidence was 10.0 per 100 (IQR = 1.7); ranging from 6.2 in 2011 in Telangana to 12.5 in 2019 in Odisha (Supplement Table S6). All the predictor variables were significantly different between HF and nHF states (Wilcoxon rank sum test; *p* < 0.001).

### Correlation between variables

**Figure 2:**
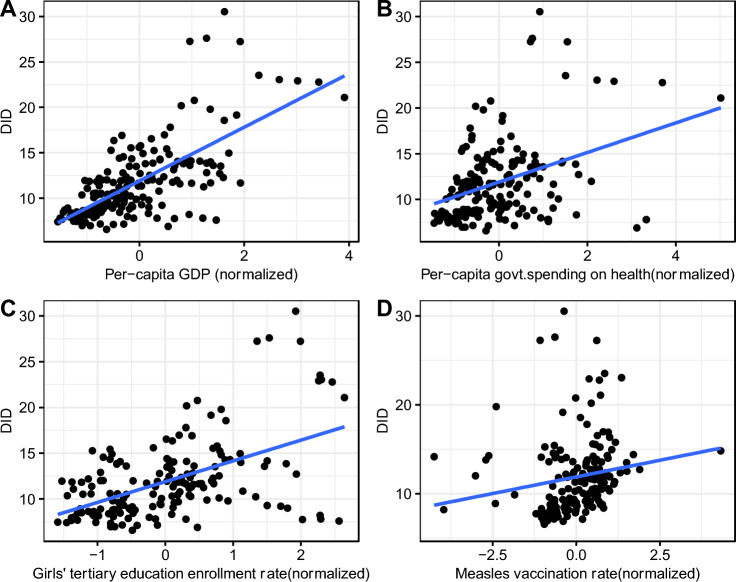
Scatterplots and superimposed best-fit lines showing associations between private-sector antibiotic consumption (DID) and (**A**) normalized per-capita GDP, (**B**) normalized government spending on health, (**C**) normalized girls’ tertiary enrollment rate, and (**D**) normalized measles vaccination rate, India, 2011–2019. *DID* defined daily doses (DDD) per 1000 population per day, *GDP* gross domestic product.

**Figure 3:**
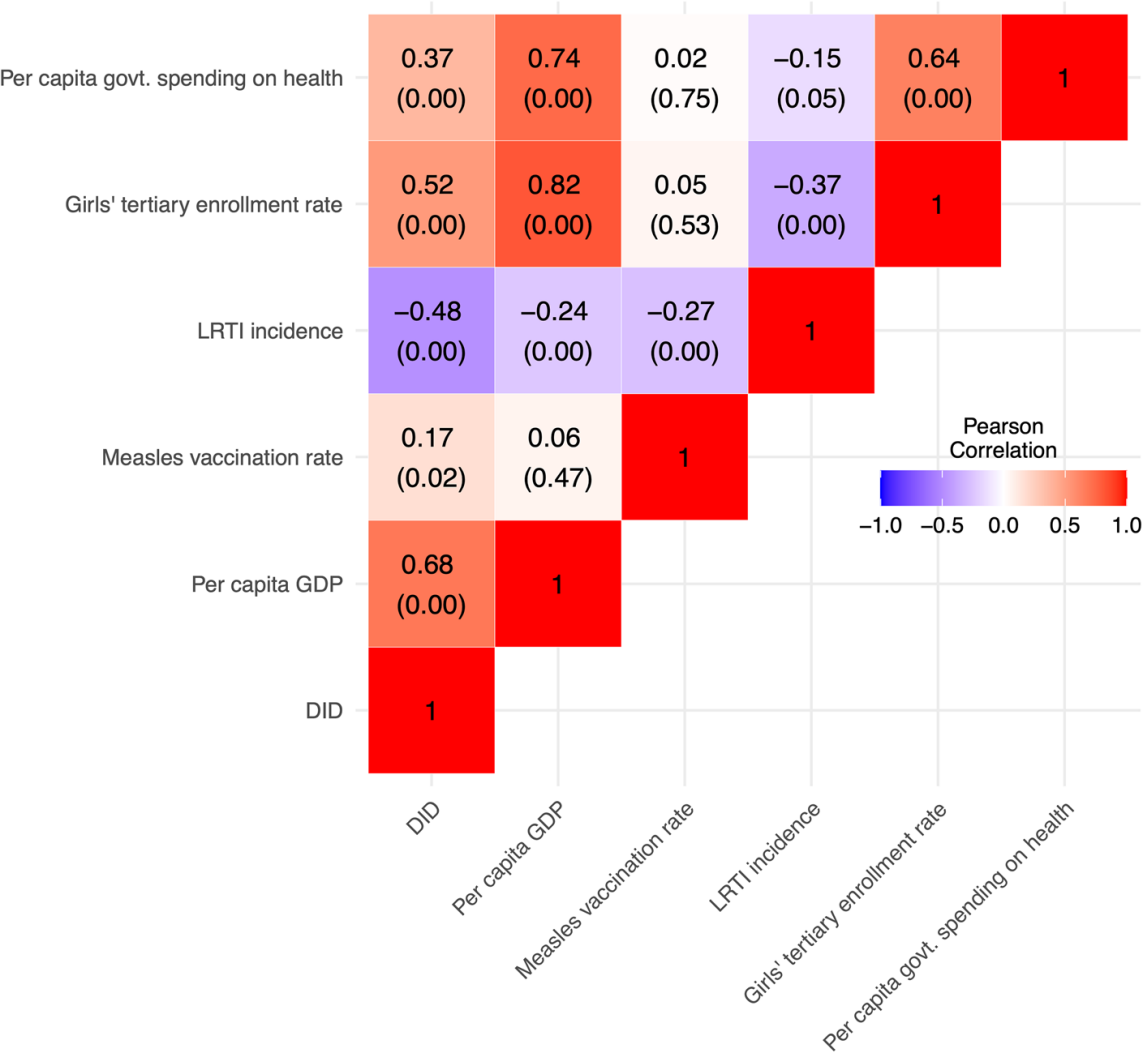
Pairwise Pearson correlation coefficients between private-sector antibiotic consumption (DID) and predictor variables, India, 2011–2019. The values inside the square boxes are the correlation coefficients and the values in parenthesis are the *p* values for corresponding coefficients. *DID* defined daily doses per 1000 population per day, *GDP*—Gross Domestic Product, in Indian Rupees (₹ in ‘000 s, at current prices); Vaccination—Measles Vaccination rate, per 1000 eligible children; LRTI—Lower Respiratory Tract Infection per 100 population; Education—Girls’ enrollment in tertiary education, %; Government spending—per capita government spending on health, ₹; Sources of data: DID—PharmaTrac, GDP—Reserve Bank of Indian report, Government spending on health—state budget documents, Measles vaccination rate—India health management information system, LRTI incidence—GBD data, IHME, Girls’ enrollment rate data —CEIC database sourced from Ministry of Education.

The antibiotic consumption showed moderate to strong positive correlation with per-capita GDP. The pairwise Pearson correlation (r) was significant (r = 0.68, *p* < 0.01). Antibiotic consumption showed moderate positive correlation with girls’ education (r = 0.52, *p* < 0.01) and government spending (r = 0.37, *p* < 0.01), and weak positive correlation with measles vaccination (r = 0.17, *p* = 0.02). Antibiotic consumption was negatively correlated with LRTI incidence (r = − 0.49, *p* < 0.01) (Figs. 2 and 3).

Table 2 shows the results of the fixed effects adjusted linear regression models with standard errors adjusted for 19 state-clusters. Model 1 without adjusting for population showed that private sector antibiotic consumption significantly increased with increase in LRTI incidence (β = 1.705, *p* = 0.001). Antibiotic consumption significantly decreased with increase in per-capita GDP (β = − 0.028, *p* = 0.006), per-capita government spending on health (β = − 1.264, *p* = 0.008), and girls’ tertiary education enrollment (β = − 0.127, *p* = 0.008) after adjusting for increase in LRTI incidence and all state-level measured and unmeasured time-invariant confounders. In absolute terms, these results translate to a decrease in the private sector antibiotic consumption by 10.22 antibiotic doses per 1000 population per year for every ₹1000 (US$12.9) increase in per-capita GDP, by 461.4 antibiotic doses per 1000 population per year with every ₹1000 increase in per-capita government health spending, and by 46.4 doses per 1000 population per year with every one percent increase in girls’ enrollment rate in tertiary education. The antibiotic consumption increased by 622.3 doses per 1000 population per year with every one percent increase in LRTI incidence. Including population changes in the model (Model 2) did not substantially change the findings.

**Table 2:** Fixed effects adjusted linear regression models for private-sector antibiotic consumption in India.

Variable	Model 1^a^			Model 2^b^	
	β [95% CIs]	SE	*p* value	β [SE]	*p* value
Population, in millions	–	–	**–**	− 0.054 [0.039]	0.186
Per capita GDP, in ₹ ‘000 s, at current prices	− 0.028 [− 0.048, − 0.009]	0.009	**0.006**	− 0.031 [0.009]	**0.003**
Per capita government spending on health, in ₹ ‘000 s	− 1.264 [− 2.155, − 0.373]	0.424	**0.008**	− 1.355 [0.443]	**0.007**
Girls’ tertiary education enrollment, %	− 0.127 [− 0.217, − 0.037]	0.043	**0.008**	− 0.133 [0.040]	**0.003**
Measles Vaccination rate, per 1000 eligible children	− 0.001 [− 0.003, 0.001]	0.001	0.158	− 0.001 [0.001]	0.163
Incidence of LRTI, per 100 population	1.705 [0.75, 2.659]	0.455	**0.001**	1.633 [0.415]	**0.001**
Number of states	19				
Number of observations	171				

Model 1 did not adjust for population changes, Model 2 adjusted for population changes.

Significant values are in [bold].

*GDP* gross domestic product, *LRTI* lower respiratory tract infection, *SE* standard error.

^a^corr(u_i, Xb) = − 0.9366; rho = 0.99; R-squared: Within = 0.4387; Between = 0.5979; Overall = 0.5182.

^b^corr (u_i, Xb) = − 0.8989; rho = 0.99; R-squared: Within = 0.4463; Between = 0.4052; Overall = 0.3441.

### Sensitivity analysis

The results of sensitivity analysis—differences across HF and nHF states—with and without adjusting for changes in populations—are shown in Table 3. Model 1 which did not adjust for population changes showed that increase in LRTI incidence was significantly associated with increased antibiotic consumption in nHF states (β = 2.137, *p* = 0.005) but the association was not significant in HF states (*p* = 0.061). Increased government health spending had a negative effect on antibiotic consumption in HF (β = − 0.872, *p* = 0.001) and nHF states (β = − 1.694, *p* = 0.044). Girls’ education enrollment (β = − 0.151, *p* = 0.009) had a significant negative effect on antibiotic consumption in nHF states only. However, we prefer to interpret these results with caution, considering the fewer sample of states in each group. Including population changes in the model (Model 2) did not substantially change the findings.

**Table 3:** Fixed effects adjusted regression models for private-sector antibiotic consumption in High Focus and non-High Focus States in India, 2011–2019.

Variable	Model 1				Model 2			
	High focus states^a^		Non-high focus states^b^		High focus states^c^		Non-high focus states ^d^	
	β [SE]	*p* value	β [SE]	*p* value	β [SE]	*p* value	β [SE]	*p* value
Population, in millions	–	–	–	**–**	− 0.022 [0.026]	0.438	− 0.286 [0.121]	**0.040**
Per capita GDP, in ₹ ‘000 s, at current prices	− 0.027 [0.015]	0.121	− 0.014 [0.012]	0.276	− 0.022 [0.015]	0.194	− 0.023 [0.010]	0.051
Per capita government spending on health, in ₹ ‘000 s	− 0.872 [0.166]	**0.001**	− 1.694 [0.736]	**0.044**	− 1.087 [0.379]	**0.024**	− 1.50 [0.682]	0.052
Girls’ education enrollment, %	− 0.054 [0.070]	0.469	− 0.151 [0.047]	**0.009**	− 0.066 [0.062]	0.314	− 0.195 [0.044]	**0.001**
Measles Vaccination rate, per 1000 children	0.001 [0.001]	0.282	− 0.002 [0.001]	0.068	0.001 [0.001]	0.258	− 0.002 [0.001]	0.098
Incidence of LRTI, per 100 population	1.286 [0.576]	0.061	2.137 [0.595]	**0.005**	1.215 [0.583]	0.076	2.236 [0.541]	**0.003**
Number of states	11		8		11		8	
Number of observations	99		72		99		72	

Model 1 did not adjust for population changes, Model 2 adjusted for population changes.

Significant values are in [bold].

*GDP* gross domestic product, *LRTI* lower respiratory tract infection, *SE* standard error.

^a^corr (u_i, Xb) = − 0.8271; rho = 0.96; R-squared: Within = 0.5501; Between = 0.2397; Overall = 0.1037.

^b^corr (u_i, Xb) = − 0.8625; rho = 0.98; R-squared: Within = 0.4647; Between = 0.2871; Overall = 0.2202.

^c^corr(u_i, Xb) = − 0.8825; rho = 0.97; R-squared: Within = 0.5548; Between = 0.2442; Overall = 0.1278.

^d^corr(u_i, Xb) = − 0.7489; rho = 0.98; R-squared: Within = 0.4890; Between = 0.1159; Overall = 0.1232.

## Discussion

Using a quasi-experimental study design, we showed that economic productivity, government health spending, and girls’ higher education strongly influence antibiotic consumption, independent of infectious disease burden, and public health interventions like vaccinations after adjusting for all measured and unmeasured time-invariant confounders. Antibiotic consumption increased by 622.3 doses per 1000 population per year with every 1% increase in LRTI incidence. The most important determinant of private antibiotic consumption was government health spending. We discuss four key findings from our analysis below.

First, overall economic productivity and growth leads to significant reduction in antibiotic consumption. Every ₹1000 (US$12.9) increase in per-capita GDP led to a decrease in private sector antibiotic consumption by 0.028 doses per 1000 population per day or 10.22 antibiotic doses per 1000 population per year. Our results agree with the study by García-Rey et al. that reported a significant negative association between GDP per capita and antibiotic use in Spain^1^. However, our results contrast with the findings by Klein et al.^26^ that reported a significant positive association between changes in per capita GDP and antibiotic consumption in LMICs, but not in HICs. Typically, with increased economic growth, the availability of pharmaceutical products in the market may improve in LMICs and may lead to improved access and consumption. However, this may not be the case with India, as the country already had a substantial pharmaceutical industry that had produced and made available antibiotics in private sector (similar to HICs) even before the major economic reforms in 1990s and the subsequent economic growth^32^. In their 2018 paper, Tamhankar et al. reported no influence of economic growth indicators on antibiotic consumption in India^33^, using data from 2000 to 2010. However, this analysis used only national level antibiotic consumption data and did not use DDD, the standard unit of antibiotic use. This contrasts with our study which analyzed state-level panel data for several years employing a rigorous fixed effects design and suggests a strong inverse causal relation between economic growth and private sector antibiotic consumption after adjusting for various factors. Our finding may be explained in part by the changes in more distal factors that influence antibiotic consumption following economic progress, for example, improved availability of drinking water, sanitation facilities, and nutrition—and not merely the improved availability of medicines in public sector—as our results were adjusted for changes in government expenditure in health.

Second, increased government spending on health had the most significant effect in reducing private sector antibiotic consumption. With every ₹1000 (US$12.9) increase in per-capita government health spending, private-sector consumption of antibiotics reduced by 461.4 antibiotic doses per 1000 population per year. The 2015 WHO Global Action Plan called for political will to make effective antibiotics available and accessible for patients, including through government spending on health as a means to reducing inappropriate use and antibiotic resistance^34^. Data from the US show that 30–75% of antibiotics prescribed in hospitals, nursing homes, doctor’s offices and emergency departments are unnecessary^35^. Considering the catastrophic financial consequences of antibiotic resistance due to an increase in hospital admissions and drug usage following inappropriate use, the return on investment in making appropriate antibiotics accessible through public systems is high^36^. However, government spending on health improves not only antibiotic availability in the public sector but may also improve vaccination services, and infection prevention and control activities including awareness programs, all of which may reduce antibiotic use. These findings support an earlier analysis of WHO antibacterial resistance surveillance report which concluded that copayment requirements for drugs in public sector can incentivize private sector to overprescribe antibiotics^16^. Further, our study adds to the literature on successful government policy interventions to reduce antibiotic use especially in countries where out-patient healthcare provision is through largely unregulated private providers^37^.

Third, increase in measles vaccination rate was not found to be associated with antibiotic consumption, consistent with what Klein et al. reported^26^. A meta-analysis in 2019 found that although the overall evidence base on the effect of vaccination on antibiotic use is poor, randomized controlled trials (RCT) mostly reported reductions in antibiotic use following vaccinations^38^. This was particularly the case with studies on pneumococcal and influenza vaccines similar to what was reported by Klugman and Black^39^. However, these papers and a 2012 review^40^ included studies mostly from HICs, and had only one very low quality evidence RCT with measles vaccine that reported no significant difference in antibiotic use.^41^ These suggest that measles vaccination rate may not be an appropriate measure to test the effect of vaccination on antibiotic use. We did not have sufficient data to include pneumococcal and influenza vaccines in our analysis, which if considered, may give a different result.

Lastly, improvement in girls’ higher education enrollment significantly reduces antibiotic consumption. With every one percent increase in girls’ enrollment rate in tertiary education, antibiotic use decreased by 46.4 doses per 1000 population per year. Our findings are in line with a recent Southeast Asia scoping review that had shown that women have better knowledge of antibiotics and were less likely to buy antibiotics over the counter^42^. A national survey from Thailand in 2017 also showed that women with higher levels of education had significantly higher chance of obtaining information on appropriate use of antibiotics^43^. A more recent meta-analysis showed that higher education was associated with 14% lower odds of any aspect of antibiotic misuse in LMICs, whereas higher education was associated with 25% higher odds of antibiotic misuse in Europe^44^.

Our analysis showed that increased public investment in health can reduce private sector antibiotic consumption which may help in reducing antibiotic resistance as observed by Collignon et al. in their analysis^45^*.* The better availability of antibiotics in the public sector especially in primary healthcare facilities may reduce the prescription from private facilities. This is critically important as most of the antibiotic use happens in the primary care^46^ particularly for self-limiting illnesses^47^.

### Strengths and limitations

The quasi-experimental design provided an opportunity to perform a ‘natural experiment’ and study the effect of some of the key factors that influence antibiotic consumption. In addition, the panel data available helped to control the effects of many measured and unmeasured confounders that may not vary substantially across time—including population and provider awareness and behavior, health infrastructure including human resources, climate factors, and population age structure and density. However, one may challenge the assumption that provider behavior and health infrastructure have not changed substantially in the 10-year period. By including government funding on health as a predictor we might have covered the infrastructure changes.

Fixed effects regression works under the assumption of strict exogeneity, meaning, we assume there is no reverse causality and no reverse feedback from past outcomes to current covariates and current outcome to future covariates. However, in our model, this assumption may be violated when we consider the relation between antibiotic use and infection. Another important limitation is that our model included only a few selected time-varying covariates and therefore might have missed out the effect of other time-varying factors—one key factor being improvement in water and sanitation facilities. Additionally, we had to limit the analysis to using the measles vaccination rate as we lacked long-term data on pneumococcal and influenza vaccines.

Our analysis is restricted to private sector data and as we could not examine changes in consumption through public sector purchases, some of our findings could be explained by a shift from the private sector (which we observed) to the public sector (which we did not observe), although this is likely to be minimal given the dominant role of the private healthcare sector in India.

## Conclusion

We examined the effect of some of the key factors determining antibiotic consumption using state level data from India from 2011 to 2019. This is the first study in the Indian context examining the determinants of antibiotic consumption. We found that economic progress (GDP), social progress (girls’ higher education), and welfare measures (government spending on health) can reduce private sector antibiotic consumption. India’s ongoing revision of its Antimicrobial Resistance National Action Plan should consider creating a robust and sustainable antibiotic consumption and use surveillance system that captures data from public and private sectors. Data on antibiotic use from both sectors will help in designing targeted stewardship programs and in assessing impact of various interventions and investments including newer vaccines and water and sanitation measures in reducing antibiotic use.

## Materials and methods

### Setting

Annual state-level private sector antibiotic consumption data across 19 Indian states from 2011 to 2019.

### Measures

#### Outcome

Per-capita private sector antibiotic consumption in Defined Daily Doses (DDD) per 1,000 Inhabitants per Day (“DID”). DDD is the assumed average maintenance dose per day for a drug used for its main indication in adults^31^. We calculated total DDDs across all antibiotics used in private sector using PharmaTrac antibiotics sale volume (strength of the product, number of “packs consumed” and “pack size”) and unit DDD values for molecules or formulation from WHO database. Then we used population figures to calculate the DIDs—the details of which are available in our previous paper^48^ and are indicated in formulae below.

$$Total\,DDDs\,Consumed=\frac{Strength*Pack\,Size*Packs\,Consumed}{DDD\,of\,Molecule/formulation}$$

$$DIDs=\frac{Total\,DDDs\,Consumed}{Population\,in\,thousands*365}$$

#### Predictors

Our independent variables are per-capita net state domestic product[NSDP], per capita government spending on health, girls’ tertiary education enrollment rate, measles vaccination coverage, and incidence of lower respiratory infections (LRTI).

### Data sources

The outcome measure (DID) was based on the analysis of PharmaTrac data, the details of which have been published previously^31,48^. Briefly, PharmaTrac gathers primary data from a panel of 9000 pharmaceutical distributors and 500,000 retailers and extrapolate the data to represent the entire private retail sector pharmaceutical sales. The PharmaTrac dataset combines data for some states and therefore the number of states (n = 19) is smaller than the actual number (n = 28). Wherever data are combinedly presented for states, we have used the combined population for our analysis. Besides data were not available for the state of Jammu and Kashmir and for union territories. The drugs dispensed through public facilities—which accounts for less than 15–20% of all drug sales in the country—are not covered by PharmaTrac.

We used the mid-year, state level population (in millions) from the National Population Commission (https://censusindia.gov.in/) (Supplement Table S1).

We gathered the data on net state domestic product (NSDP) from the national statistical office, Reserve Bank of India (https://www.rbi.org.in/). We calculated annual per-capita NSDP, in Indian Rupees (INR, [₹], in ‘000 s, at current prices) for the years 2011–2019.

We used the annual budget documents of state governments to gather the per-capita amount (INR, [₹], in ‘000 s, at current prices) spent by the state governments on health every year.

Girls’ tertiary enrollment rate is the number of girls in tertiary level education as percent of all girls who have finished secondary school in the previous 5 years^49^. We sourced the data from Ministry of Education, Government of India using the search option in the CEIC website (https://www.ceicdata.com/).

Measles immunization rate from India's health management information system (HMIS) standard report (Standard Reports/12 ~ H. RCH Reports) provided state-wise tabulated data on immunizations and is used for Global burden of disease estimations. We retrieved this data in excel format from the HMIS portal where the measles and measles-rubella (MR) vaccination rate are available under the item codes 9.2.1 and 9.2.2 (https://hmis.nhp.gov.in/). The vaccination coverage is expressed as the number of children up to one year of age who received at least one dose of measles/MR vaccine during the current year for every 1000 eligible children.

We retrieved the state level estimates of the incidence of LRTIs *for both sex for all ages* for the years 2011–2019 from the Global Burden of Diseases (GBD) data from IHME database (https://vizhub.healthdata.org/^50^, and used the incidence per 100 population for analysis (referred to as LRTI incidence).

### Ethical consideration

This research did not involve any human subjects and the data used for the analysis did not include patient identifiers, therefore this study did not require review by an institutional review board.

### Study design

We used fixed effects regression, a quasi-experimental causal inference design to answer our question, using a cross-sectional time-series (panel) data. The empirical data sources are described above. There are numerous measured and unmeasured confounding factors that may affect the relation between antibiotic consumption and these predictors. For example, the state population-age composition may affect the respiratory infection rate and antibiotic use. Similarly, health service availability, doctor-population ratio, and health attitude, perception, and awareness levels may affect vaccination rate and antibiotic consumption. Further, various cultural and social characteristics may affect girls’ education and antibiotic consumption. However, these confounding variables are largely time-invariant, i.e., they remain largely unchanged during the study period. We used the longitudinal nature of the data to control for confounding effect of these time-invariant measured and unmeasured factors by using a fixed effects regression method as discussed below.

#### Fixed effects regression model

Let ${y}_{it}$ denotes the DID for $i$*-*th state in $t$*-*th year, $\alpha$ represents the intercept, $\beta$ denotes the vector of regression coefficients for the time-varying predictors of interest represented by vector ${x}_{it}$, and $\gamma$ denotes the vector of regression coefficients of time-invariant observed confounders represented by vector ${z}_{i}$. Consider that we measured the variables at two years (T = 2). The equations to calculate the DID for $i$ *-*th state for year 1 and 2 are:

1 $${y}_{i1 }=\alpha +\beta {x}_{i1}+ \gamma {z}_{i}+ {u}_{i}+ {\varepsilon }_{i1}$$

2 $${y}_{i2 }=\alpha +\beta {x}_{i2}+ \gamma {z}_{i}+ {u}_{i}+ {\varepsilon }_{i2}$$

where ${u}_{i}$ is the error term that represents the combined effect on *y* of all unobserved confounders that are constant over time; and ${\varepsilon }_{it}$ is the "idiosyncratic error"— random error that changes over time and across states. Therefore, there is a different ${{\text{u}}}_{{\mathrm{I}}}$ for every state and that remains the same across the years, and a different set of ${\upvarepsilon }_{{\mathrm{it}}}$ for each state that varies from year to year.

Further, we cannot assume statistical independence of ${u}_{i}$ and ${x}_{it}$ due to confounding. However, when we subtract Eq. (2) from Eq. (1), we get the following first-difference equation without ${u}_{i}$:

3 $$\Delta y= \beta\Delta {x}_{i}+\Delta {\varepsilon }_{i}$$

where $\Delta$ is the difference score. When it comes to multiple years, first we calculate the mean value of outcome (antibiotic use) and each predictor variable over time for each state as below.

$${\overline{y} }_{i}=\frac{1}{{n}_{i}} \sum_{t}{y}_{it}$$

$${\overline{x} }_{i}=\frac{1}{{n}_{i}} \sum_{t}{x}_{it}$$

where ${n}_{i}$ is the number of measurements for state $i$ .

Second, we subtract the state-specific means from the observed values for each variable.

$${y}_{it}^{*}={y}_{it }-{\overline{y} }_{i}$$

$${x}_{it}^{*}={x}_{it }-{\overline{x} }_{i}$$

$${\varepsilon }_{it}^{*}={e}_{it }-{\overline{\varepsilon }}_{i}$$

Finally, we regress ${y}^{*}$ on ${x}^{*}$ as:

$${y}_{it}^{*}= \beta {x}_{it}^{*}+ {\varepsilon }_{it}^{*}$$

With this, we can estimate the change in DID as a function of changes in values of predictors after adjusting all time-invariant observed (${z}_{i}$) and unobserved (${u}_{i}$) confounders (*fixed effect*) that could influence the coefficient, $\beta$.

Our final fixed effects model was:

$${DID}_{it}^{*}= {\beta }_{1}{(NSDP}_{it }^{*})+ {\beta }_{2}{(per-capita\,government\,spending\,on\,health }_{it }^{*})+ {\beta }_{3}{(girl{s}{\prime}tertiary\,education\,enrollment\,rate}_{it }^{*})+{ {\beta }_{4}{(measles\,vaccination\,coverage}_{it }^{*})+{\beta }_{5}{(LRTI\,incidence}_{it }^{*})+ \varepsilon }_{it}^{*}$$

### Statistical analysis

First, we prepared a spreadsheet with the state-wise annual DID data as reported previously^31,48^. Then, we collated and organized predictor variables data in a second spreadsheet. We then merged these two sheets and created the final panel data for analysis using STATA software version 17.0 (Stata Corp LP, 2021).

We conducted descriptive analysis of antibiotic use (DID) and independent variables using median, and interquartile range, and plotted line graphs to visually inspect the changes in the outcome and predictor variables over years. We presented the summary of these variables in tables and compared the values across HF and nHF states. We used Wilcoxon rank sum test to assess statistically significant differences as the data were not normally distributed and the sample sizes were small. Then we conducted pair-wise correlation of each predictor with the dependent variable and calculated pairwise Pearson correlation coefficients. A *p* value less than 0.05 was considered statistically significant in all analysis. We also used scatter plots to visualize the relationship between the pairs using normalized values for independent variables. We used R software version 4.1.1 (R Core Team, 2020) and packages *tidyverse* and *ggplot2* for the analysis.

Then we created linear fixed effects and random effects regression models using the *xtreg* command in STATA by specifying the state ID as the variable to identify the record for each state. We tested for autocorrelation in our data using Wooldridge test which confirmed first-order autocorrelation (F (1,18) = 14.4, *p* = 0.001). The overall F-test showed that the fixed effects are non-zero (F (13,18) = 18.36, *p* < 0.001); and that a pooled ordinary least square regression will be biased. The unobserved heterogeneity ($${u}_{i})$$ strongly correlated with explanatory variables ($${x}_{it})$$ − (Cov ($${x}_{it}$$, $${u}_{i})$$ = − 0.94)—evidence of confounding, supporting the choice of fixed effects model. Further, the Hausman test verified that the random effects model will be biased ($${\chi }^{2}$$ = 115.4, *p* < 0.001) and confirmed our choice of fixed effects model. Finally, we used the *vce (robust)* command to get cluster robust standard errors to control for heteroskedasticity and within panel serial correlation. Additionally, errors were clustered by state to account for high serial correlation. Finally, we conducted sensitivity analyses—by conducting the fixed effects regression analysis separately for high-focus (HF) and non-high-focus (nHF) states—with and without population as a predictor in the model. A *p* value less than 0.05 was considered statistically significant for all statistical tests.

### Supplementary Information

Supplementary Tables.

## Data Availability

The PharmaTrac data used in this study are available from the AIOCD Pharma soft tech AWACS Pvt. Ltd. Restrictions apply to the availability of these data, which were used under license for this study. Permission can be obtained at https://aiocdawacs.com. All the remaining data are available from public sources mentioned in the paper, and the state-wise year-wise values are given in supplement tables.
