# Supplementary material for: Determinants of private-sector antibiotic consumption in India: findings from a quasi-experimental fixed-effects regression analysis using cross-sectional time-series data, 2011–2019
Source: Sci Rep. 2024 Feb 29;14:5052. doi: 10.1038/s41598-024-54250-2 (PMC10904839; doi:10.1038/s41598-024-54250-2)
Supplement: Supplementary file 1 — Supplementary Tables. [file 41598_2024_54250_MOESM1_ESM.docx]

Supplementary Materials for

**Determinants of antibiotic consumption: Findings from a quasi-experimental study using cross-sectional time-series data from India, 2011- 2019**

Shaffi Fazaludeen Koya*, Senthil Ganesh, Sakthivel Selvaraj, Veronika J. Wirtz, Sandro Galea, Peter C. Rockers

*Corresponding author. Email: [fmshaffi@bu.edu](mailto:fmshaffi@bu.edu)

**This file includes:**

Tables S1 to S7

| **Table S1: Population in millions** | | | | | | | | | |
| --- | --- | --- | --- | --- | --- | --- | --- | --- | --- |
| States/Year | 2011 | 2012 | 2013 | 2014 | 2015 | 2016 | 2017 | 2018 | 2019 |
| Andhra Pradesh | 49.6 | 49.9 | 50.3 | 50.7 | 51 | 51.4 | 51.7 | 51.9 | 52.2 |
| Delhi | 16.8 | 17.2 | 17.5 | 17.9 | 18.3 | 18.7 | 19.1 | 19.4 | 19.8 |
| Gujarat | 61 | 62 | 63 | 64 | 65 | 65.9 | 66.9 | 67.9 | 68.9 |
| Haryana | 25.4 | 25.8 | 26.2 | 26.6 | 27 | 27.5 | 27.9 | 28.3 | 28.7 |
| Karnataka | 61.1 | 61.7 | 62.3 | 63 | 63.6 | 64.2 | 64.8 | 65.3 | 65.8 |
| Kerala | 33.5 | 33.7 | 33.9 | 34.2 | 34.4 | 34.6 | 34.8 | 35 | 35.2 |
| Maharashtra | 112.4 | 113.6 | 114.9 | 116.2 | 117.5 | 118.7 | 119.9 | 121 | 122.2 |
| Punjab | 35.7 | 36 | 36.4 | 36.7 | 37.1 | 37.4 | 37.7 | 38 | 38.3 |
| Tamil Nadu | 73.8 | 74.3 | 74.8 | 75.4 | 75.9 | 76.4 | 76.8 | 77.2 | 77.6 |
| Telangana | 35 | 35.3 | 35.6 | 35.9 | 36.2 | 36.5 | 36.7 | 37 | 37.2 |
| West Bengal | 91.3 | 92 | 92.8 | 93.6 | 94.3 | 95.1 | 95.7 | 96.3 | 96.9 |
| Bihar | 104.1 | 106.1 | 108.1 | 110.1 | 112.2 | 114.2 | 116 | 117.7 | 119.5 |
| Chhattisgarh | 25.5 | 26 | 26.4 | 26.8 | 27.2 | 27.6 | 28 | 28.3 | 28.7 |
| Jharkhand | 33 | 33.6 | 34.1 | 34.7 | 35.2 | 35.8 | 36.3 | 36.9 | 37.4 |
| Madhya Pradesh | 72.6 | 73.9 | 75.1 | 76.3 | 77.6 | 78.8 | 79.9 | 81.1 | 82.2 |
| Northeast | 45.8 | 46.3 | 46.9 | 47.4 | 48 | 48.5 | 49.1 | 49.6 | 50.1 |
| Odisha | 42 | 42.4 | 42.8 | 43.2 | 43.6 | 44 | 44.3 | 44.7 | 45 |
| Rajasthan | 68.5 | 69.7 | 70.8 | 72 | 73.1 | 74.2 | 75.2 | 76.3 | 77.3 |
| Uttar Pradesh | 216.8 | 220.2 | 223.7 | 227.1 | 230.6 | 234 | 237.1 | 240.3 | 243.4 |

| **Table S2: Per-capita GDP in '000 Rupees** | | | | | | | | | |
| --- | --- | --- | --- | --- | --- | --- | --- | --- | --- |
| States/Year | 2011 | 2012 | 2013 | 2014 | 2015 | 2016 | 2017 | 2018 | 2019 |
| Andhra Pradesh | 69 | 74.7 | 82.9 | 93.9 | 108 | 120.7 | 138.3 | 152.3 | 168.5 |
| Delhi | 185 | 205.6 | 227.9 | 247.2 | 270.3 | 295.6 | 318.3 | 344.4 | 376.2 |
| Gujarat | 87.5 | 102.8 | 113.1 | 127 | 139.3 | 156.3 | 177 | 197.5 | 213.9 |
| Haryana | 106.1 | 121.3 | 137.8 | 147.4 | 165 | 185 | 210.6 | 226.4 | 247.6 |
| Karnataka | 90.3 | 102.3 | 118.8 | 130 | 148.1 | 169.9 | 186.4 | 205.7 | 223.2 |
| Kerala | 97.9 | 110.3 | 123.4 | 135.5 | 148.1 | 166.2 | 183.3 | 205.7 | 221.9 |
| Maharashtra | 99.6 | 112.1 | 125.3 | 132.8 | 146.8 | 163.7 | 172.7 | 187.1 | 202.1 |
| Punjab | 122.3 | 137.4 | 153.6 | 160.8 | 174.4 | 190.5 | 210.2 | 227.6 | 242.8 |
| Tamil Nadu | 100.6 | 111.6 | 125.4 | 134.3 | 150.6 | 166 | 184.1 | 201 | 217.7 |
| Telangana | 91.1 | 101 | 112.2 | 124.1 | 140.8 | 159.4 | 179.4 | 210.6 | 233.3 |
| West Bengal | 51.5 | 58.2 | 65.9 | 68.9 | 76 | 82.3 | 91.4 | 103.9 | 113.2 |
| Bihar | 21.8 | 24.5 | 26.9 | 28.7 | 30.4 | 34 | 36.9 | 40.7 | 45.1 |
| Chhattisgarh | 55.2 | 60.8 | 69.9 | 72.9 | 73 | 83.3 | 89.7 | 98.3 | 105.1 |
| Jharkhand | 41.3 | 47.4 | 50 | 57.3 | 52.8 | 60 | 67.5 | 75.4 | 77.7 |
| Madhya Pradesh | 38.5 | 44.8 | 51.8 | 55.7 | 62.1 | 74.3 | 82 | 90.5 | 103.3 |
| Northeast | 66.4 | 73.2 | 82.8 | 93.8 | 103.6 | 114.3 | 133.7 | 144.5 | 158.2 |
| Odisha | 48.5 | 55.1 | 61.3 | 64.2 | 66 | 79.2 | 89.4 | 100.8 | 110.4 |
| Rajasthan | 57.2 | 63.7 | 69.5 | 76.4 | 83.4 | 91.9 | 98.2 | 107.9 | 115.5 |
| Uttar Pradesh | 66.2 | 74.7 | 83.2 | 89.2 | 97.5 | 107.2 | 118.7 | 127.1 | 134.3 |

GDP: Gross Domestic Product

| **Table S3: Per-capita government spending on health in Indian Rupees** | | | | | | | | | |
| --- | --- | --- | --- | --- | --- | --- | --- | --- | --- |
| States/Year | 2011 | 2012 | 2013 | 2014 | 2015 | 2016 | 2017 | 2018 | 2019 |
| Andhra Pradesh | 1110.0 | 1234.2 | 1308.7 | 990.9 | 974.9 | 1111.4 | 1236.8 | 1424.5 | 2223.3 |
| Delhi | 1470.7 | 1492.6 | 1582.9 | 1917.5 | 1895.2 | 2277.2 | 2483.8 | 3070.6 | 3777.8 |
| Gujarat | 634.9 | 827.6 | 920.6 | 950.3 | 1038.8 | 1109.2 | 1272.4 | 1527.5 | 1560.9 |
| Haryana | 575.3 | 724.0 | 798.7 | 802.6 | 906.7 | 1250.1 | 1211.9 | 1583.1 | 1749.6 |
| Karnataka | 650.4 | 746.7 | 832.1 | 884.1 | 891.8 | 1003.0 | 1253.5 | 1510.0 | 1473.2 |
| Kerala | 948.2 | 1098.9 | 1211.7 | 1188.3 | 1312.9 | 1622.7 | 1861.1 | 2018.0 | 2053.6 |
| Maharashtra | 586.0 | 699.6 | 754.0 | 730.0 | 817.7 | 1024.6 | 1015.7 | 1332.7 | 1303.2 |
| Punjab | 651.5 | 757.4 | 761.2 | 739.2 | 805.2 | 988.3 | 900.0 | 1126.9 | 1133.8 |
| Tamil Nadu | 723.4 | 842.2 | 1010.4 | 989.6 | 1106.1 | 1164.8 | 1424.2 | 1679.0 | 1687.2 |
| Telangana | 1110.0 | 1234.2 | 1308.7 | 690.8 | 1020.3 | 1524.0 | 1370.3 | 1849.3 | 1383.1 |
| West Bengal | 500.9 | 519.0 | 613.4 | 632.8 | 779.5 | 813.4 | 925.5 | 976.1 | 1003.8 |
| Bihar | 302.7 | 343.7 | 365.6 | 309.1 | 395.4 | 657.1 | 533.1 | 681.6 | 766.1 |
| Chhattisgarh | 585.6 | 657.1 | 854.9 | 842.5 | 967.0 | 1480.8 | 1433.6 | 1809.2 | 1717.5 |
| Jharkhand | 475.3 | 386.6 | 468.8 | 439.9 | 592.1 | 810.5 | 786.6 | 1087.2 | 1108.7 |
| Madhya Pradesh | 393.3 | 596.5 | 644.4 | 563.4 | 678.4 | 771.8 | 931.7 | 960.1 | 1276.7 |
| Northeast | 1097.6 | 1151.6 | 1182.2 | 1083.1 | 1211.3 | 1744.3 | 2027.9 | 2874.7 | 2759.7 |
| Odisha | 475.7 | 570.8 | 649.3 | 709.2 | 819.9 | 1089.5 | 1111.9 | 1373.1 | 1511.8 |
| Rajasthan | 604.6 | 689.3 | 838.8 | 858.6 | 1028.1 | 1105.2 | 1328.9 | 1595.1 | 1687.5 |
| Uttar Pradesh | 448.7 | 591.6 | 599.7 | 589.1 | 636.3 | 778.1 | 805.6 | 984.4 | 1123.4 |

| **Table S4: Girls' tertiary education enrollment rate, percentage** | | | | | | | | | |
| --- | --- | --- | --- | --- | --- | --- | --- | --- | --- |
| States/Year | 2011 | 2012 | 2013 | 2014 | 2015 | 2016 | 2017 | 2018 | 2019 |
| Andhra Pradesh | 26.4 | 23.6 | 26.7 | 27.3 | 26.8 | 28.4 | 27.1 | 29 | 32.2 |
| Delhi | 38.9 | 40.7 | 44.6 | 45.3 | 48.2 | 48.4 | 48 | 50 | 51.8 |
| Gujarat | 14.6 | 16.2 | 17.2 | 17.5 | 18.2 | 17.2 | 18.2 | 18.7 | 19.6 |
| Haryana | 27.7 | 26.5 | 25.9 | 27.4 | 26.3 | 29.6 | 30.7 | 32.4 | 32.5 |
| Karnataka | 22.6 | 24.5 | 25.6 | 26 | 25.9 | 26.6 | 28.5 | 29.4 | 32.7 |
| Kerala | 25.6 | 25.7 | 28.6 | 33.3 | 34.9 | 40 | 40.4 | 43.2 | 44.7 |
| Maharashtra | 24.2 | 20.6 | 23.9 | 25.6 | 27.5 | 28.1 | 29.5 | 30.3 | 31 |
| Punjab | 23.6 | 25.5 | 27.4 | 28.4 | 28.5 | 30.1 | 33.6 | 34.3 | 32.1 |
| Tamil Nadu | 36.8 | 38.7 | 40.4 | 43.4 | 42.4 | 45.6 | 48.2 | 48.3 | 51 |
| Telangana | 26.4 | 29.3 | 32.4 | 33 | 33.4 | 33.6 | 34.2 | 36.5 | 36.4 |
| West Bengal | 10.9 | 11.8 | 13.2 | 14.4 | 15.8 | 16.2 | 17.2 | 17.6 | 18.7 |
| Bihar | 10.8 | 11.6 | 11.5 | 12.4 | 12.6 | 12.8 | 11.5 | 12 | 13 |
| Chhattisgarh | 10.1 | 11.6 | 13 | 14 | 14.5 | 15.8 | 18.3 | 19.2 | 19.6 |
| Jharkhand | 9.5 | 11.9 | 12.4 | 14.8 | 14.8 | 17 | 17.6 | 18.7 | 20.9 |
| Madhya Pradesh | 14.6 | 15.1 | 17.3 | 17.3 | 17.9 | 19 | 20.5 | 21.2 | 24.2 |
| Northeast | 19.9 | 20.5 | 23.5 | 24.7 | 24.6 | 26.5 | 26.3 | 28.4 | 30.1 |
| Odisha | 14.9 | 14 | 14.8 | 15.8 | 17.8 | 18.9 | 20.1 | 20 | 20.3 |
| Rajasthan | 15.5 | 14.7 | 17.7 | 17.8 | 18.5 | 19.2 | 20.6 | 23 | 23.9 |
| Uttar Pradesh | 24.8 | 27.2 | 28.9 | 29.1 | 28.9 | 29.2 | 31.5 | 33.3 | 34.6 |

| **Table S5: Measles vaccination coverage, per 1000 eligible children** | | | | | | | | | |
| --- | --- | --- | --- | --- | --- | --- | --- | --- | --- |
| States/Year | 2011 | 2012 | 2013 | 2014 | 2015 | 2016 | 2017 | 2018 | 2019 |
| Andhra Pradesh | 1017 | 1012 | 963 | 930 | 976 | 924 | 991.5 | 1059 | 1017 |
| Delhi | 753 | 809 | 844 | 968 | 998 | 1062 | 1020 | 978 | 982 |
| Gujarat | 771 | 803 | 812.3 | 829.3 | 834 | 806.7 | 829.35 | 852 | 786.3 |
| Haryana | 1001 | 983 | 881 | 909 | 927 | 877 | 896 | 915 | 946 |
| Karnataka | 953 | 978 | 928 | 932 | 954 | 810 | 886.5 | 963 | 960 |
| Kerala | 1107 | 1081.5 | 1131 | 1082.5 | 1008 | 986 | 976.75 | 967.5 | 944.5 |
| Maharashtra | 1017 | 1014 | 1036 | 994 | 984 | 969 | 969.5 | 970 | 996 |
| Punjab | 953 | 970.5 | 992.5 | 931 | 947 | 888 | 897 | 906 | 839 |
| Tamil Nadu | 800.7 | 836 | 841.7 | 807.3 | 856.3 | 737.3 | 777.3 | 817.3 | 772 |
| Telangana | 1017 | 1012 | 963 | 1039 | 980 | 1439 | 1225 | 1011 | 1025 |
| West Bengal | 967 | 1033 | 987 | 1012 | 974 | 1009 | 990 | 971 | 975 |
| Bihar | 821 | 779 | 776 | 799 | 932 | 982 | 892 | 802 | 952 |
| Chhattisgarh | 864 | 856 | 823 | 851 | 907 | 895 | 918 | 941 | 951 |
| Jharkhand | 856 | 849 | 805 | 814 | 893 | 994 | 919 | 844 | 982 |
| Madhya Pradesh | 846 | 790 | 781 | 758 | 765 | 806 | 787 | 768 | 914 |
| Northeast | 886.8 | 943.5 | 924.9 | 881.1 | 864.9 | 857.1 | 812.05 | 767 | 840.6 |
| Odisha | 889 | 946 | 861 | 881 | 851 | 839 | 864.5 | 890 | 856 |
| Rajasthan | 795 | 792 | 815 | 791 | 788 | 808 | 804 | 800 | 755 |
| Uttar Pradesh | 916 | 900 | 953.5 | 885.5 | 922 | 885.5 | 923 | 960.5 | 961 |

| **Table S6: Lower respiratory tract infection incidence, per 100 population** | | | | | | | | | |
| --- | --- | --- | --- | --- | --- | --- | --- | --- | --- |
| States/Year | 2011 | 2012 | 2013 | 2014 | 2015 | 2016 | 2017 | 2018 | 2019 |
| Andhra Pradesh | 7.42 | 7.48 | 7.56 | 7.66 | 7.79 | 8.29 | 8.83 | 9.01 | 9.11 |
| Delhi | 7.96 | 7.87 | 7.77 | 7.7 | 7.71 | 8.07 | 8.49 | 8.65 | 8.73 |
| Gujarat | 10.09 | 10.05 | 10.01 | 9.99 | 10.03 | 10.57 | 11.18 | 11.36 | 11.41 |
| Haryana | 9.09 | 9.03 | 8.95 | 8.91 | 8.93 | 9.34 | 9.8 | 9.96 | 10.03 |
| Karnataka | 8.36 | 8.4 | 8.45 | 8.51 | 8.6 | 9.13 | 9.68 | 9.82 | 9.84 |
| Kerala | 9.39 | 9.47 | 9.55 | 9.67 | 9.84 | 10.62 | 11.44 | 11.67 | 11.76 |
| Maharashtra | 10.76 | 10.68 | 10.59 | 10.54 | 10.58 | 11.19 | 11.87 | 12.12 | 12.28 |
| Punjab | 8.51 | 8.65 | 8.81 | 8.98 | 9.17 | 9.58 | 9.97 | 10.14 | 10.25 |
| Tamil Nadu | 8.74 | 8.65 | 8.53 | 8.44 | 8.46 | 8.9 | 9.39 | 9.54 | 9.58 |
| Telangana | 6.2 | 6.22 | 6.26 | 6.31 | 6.36 | 6.38 | 6.42 | 6.48 | 6.53 |
| West Bengal | 10.05 | 9.9 | 9.73 | 9.61 | 9.59 | 10.02 | 10.52 | 10.68 | 10.76 |
| Bihar | 10.77 | 10.51 | 10.24 | 10 | 9.85 | 9.97 | 10.18 | 10.27 | 10.31 |
| Chhattisgarh | 9.51 | 9.38 | 9.24 | 9.12 | 9.05 | 9.15 | 9.31 | 9.39 | 9.43 |
| Jharkhand | 10.76 | 10.58 | 10.39 | 10.24 | 10.17 | 10.4 | 10.71 | 10.86 | 10.92 |
| Madhya Pradesh | 10.82 | 10.68 | 10.54 | 10.42 | 10.36 | 10.65 | 11.02 | 11.18 | 11.24 |
| Northeast | 10.11 | 10 | 9.88 | 9.8 | 9.8 | 10.16 | 10.57 | 10.73 | 10.81 |
| Odisha | 11.57 | 11.48 | 11.37 | 11.28 | 11.28 | 11.64 | 12.09 | 12.32 | 12.46 |
| Rajasthan | 10.16 | 10.13 | 10.11 | 10.11 | 10.14 | 10.55 | 11.02 | 11.15 | 11.15 |
| Uttar Pradesh | 11.13 | 11 | 10.87 | 10.77 | 10.74 | 11.05 | 11.44 | 11.59 | 11.62 |

**Table S7. Fixed effects adjusted regression models for antibiotic use in HF and nHF states in India**

| Variable | HF states^1^ | | | nHF states^2^ | |
| --- | --- | --- | --- | --- | --- |
|  | β ( SE ) | p-value | β ( SE) | | p-value |
| Per capita GDP, in ₹ ‘000s, at current prices | –0.027  (0.015) | 0.121 | –0.014  (0.012) | | 0.276 |
| Per capita government spending on health, in ₹ ‘000s | –0.872  (0.166) | **0.001** | –1.694  (0.736) | | **0.044** |
| Girl tertiary education enrollment, % | –0.054  (0.070) | 0.469 | –0.151  (0.047) | | **0.009** |
| Measles Vaccination rate, per 1000 children | 0.001  (0.001) | 0.282 | –0.002  (0.001) | | 0.068 |
| Incidence of LRTI, per 100 population | 1.286  (0.576) | 0.061 | 2.137  (0.595) | | **0.005** |
| Number of states | 11 | | | 8 | |
| Number of observations | 99 | | | 72 | |

GDP —Gross Domestic Product; LRTI—Lower Respiratory Tract Infection; HF—high focus; nHF—non-high focus; SE—Standard error

^1^corr(u_i, Xb) = –0.8271, rho= 0.96;

R-squared: Within = 0.5501 Between = 0.2397 Overall = 0.1037

^2^corr(u_i, Xb) = –0.8625, rho= 0.98;

R-squared: Within = 0.4647 Between = 0.2871 Overall = 0.2202
